# Deep learning and body composition model for predicting postoperative complications in colorectal cancer

**DOI:** 10.3389/fmedt.2026.1817439

**Published:** 2026-06-29

**Authors:** Ding Ding, Ran Xuan, Rui Li

**Affiliations:** Department of General Surgery, The Third Affiliated Hospital of Anhui Medical University (The First People’s Hospital of Hefei), Hefei, Anhui, China

**Keywords:** automatic segmentation, body composition, colorectal cancer, deep learning, machine learning, postoperative complications

## Abstract

**Background:**

Colorectal cancer (CRC) is the third most commonly diagnosed malignancy worldwide and remains a leading cause of cancer-related mortality. Surgical resection remains the cornerstone of curative treatment for CRC; however, postoperative complications, including anastomotic leakage, infections, and thromboembolic events, continue to substantially affect patient prognosis. These complications are associated with prolonged hospitalization, increased healthcare expenditures, delayed postoperative recovery, and elevated mortality risk. Therefore, the accurate identification of patients at high risk for postoperative complications is of considerable clinical importance for optimizing perioperative management and improving surgical outcomes.

**Methods:**

A total of 154 patients were retrospectively enrolled, including 99 patients in the training cohort and 55 patients in the external validation cohort. Abdominal CT images at the L1-L5 levels were automatically segmented using a pretrained DeepLabv3-ResNet101 model implemented on the Onekey platform, followed by manual correction to ensure segmentation accuracy. Visceral adipose tissue, subcutaneous adipose tissue, skeletal muscle, and intramuscular adipose tissue were segmented, and body composition indices (VAT_h, SAT_h, SMA_h, and IMAT_h) were subsequently calculated after normalization by patient height. Deep learning features were extracted from CT images using a pretrained 3D ResNet-18 model. Feature selection and model development were conducted independently. Random forest, logistic regression, and extremely randomized trees combined with recursive feature elimination were applied for feature selection. Subsequently, logistic regression, random forest, support vector machine (SVM), k-nearest neighbors (KNN), XGBoost, and LightGBM algorithms were constructed and compared for predictive performance.

**Results:**

Univariate and multivariate analyses identified BMI, SMA_h, SAT_h, and VAT_h as independent risk factors for postoperative complications. Among all predictive models, the combined model incorporating the deep learning score (DL-score) achieved the best performance, with AUC values of 0.944 and 0.855 in the training and validation cohorts, respectively. DeLong test results further demonstrated that both the deep learning model and the combined model significantly outperformed the clinical and body composition models, whereas the combined model provided superior discrimination in identifying high-risk patients.

## Introduction

Colorectal cancer (CRC) is one of the most prevalent malignancies worldwide and remains a major cause of cancer-related mortality. According to the 2022 Global Cancer Statistics (GLOBOCAN), CRC ranks as the third most commonly diagnosed cancer globally and the second leading cause of cancer-related death (1). Although surgical resection remains the core treatment for CRC, postoperative complications continue to be a significant factor affecting patient prognosis (2). Perioperative complications, including anastomotic leakage, infections, and thromboembolic events, are associated with prolonged hospitalization, increased healthcare expenditures, delayed postoperative recovery, and elevated mortality risk (3). Therefore, the accurate identification of patients at high risk for postoperative complications is of considerable clinical importance for optimizing perioperative management and improving surgical outcomes.

Several clinical variables, including elevated C-reactive protein levels, prolonged operative time, hypoalbuminemia, and intraoperative blood loss, have been identified as independent predictors of postoperative complications (4, 5). However, these conventional clinical indicators often lack sufficient specificity and predictive robustness, highlighting the need for more reliable and individualized biomarkers for perioperative risk stratification.

In recent years, body composition analysis has emerged as a valuable tool in assessing the risk of postoperative complications in CRC patients (6). In particular, CT-based body composition analysis enables quantitative assessment of adipose tissue distribution and skeletal muscle mass and has demonstrated substantial value in predicting postoperative outcomes. Previous studies have shown that increased visceral adipose tissue (VAT) is associated with a higher incidence of postoperative complications (7). Moreover, reduced skeletal muscle mass, an objective indicator of frailty and impaired physiological reserve, has been closely associated with delayed postoperative recovery and increased complication rates (8). In CRC patients, sarcopenia is considered a significant independent risk factor for postoperative complications (9). Despite its clinical utility, conventional CT-based body composition analysis frequently relies on manual segmentation, which is labor-intensive, time-consuming, and susceptible to interobserver variability.

With the rapid advancement of artificial intelligence technologies, deep learning-based automated segmentation has substantially improved the efficiency and reproducibility of CT-based body composition analysis. Deep learning models enable rapid and accurate quantification of body composition while reducing human-related bias, thereby providing a more reliable tool for preoperative risk assessment. In the present study, the DeepLabv3-ResNet101 model was employed for the automatic segmentation of visceral adipose tissue, subcutaneous adipose tissue, skeletal muscle, and intramuscular adipose tissue on abdominal CT images. Subsequently, a pretrained 3D ResNet-18 model was used to extract deep learning features from CT images. Compared with traditional handcrafted feature-based approaches, deep learning models possess superior capability in capturing complex and high-dimensional imaging patterns, thereby enabling more comprehensive characterization of tissue heterogeneity and potentially improving predictive performance.

Previous studies have attempted to combine preoperative clinical characteristics with body composition analysis to predict postoperative complications in patients with CRC (10–12). More recently, multimodal prognostic models integrating advanced imaging modalities and deep learning strategies have demonstrated promising predictive capabilities (13–15). Nevertheless, many of these approaches involve substantial methodological complexity and require extensive multimodal data, which may limit their clinical applicability and generalizability. To date, few studies have integrated deep learning features with clinical variables and CT-derived body composition metrics to establish a comprehensive predictive model for postoperative complications in CRC patients.

Therefore, the present study aimed to develop and validate a combined predictive model integrating deep learning-derived features, clinical variables, and body composition parameters for the prediction of postoperative complications in patients with CRC. By providing individualized preoperative risk assessment, this model may facilitate clinical decision-making, optimize perioperative management strategies, reduce complication rates, and ultimately improve patient prognosis.

## Methods

### Clinical information

**Figure 1:**
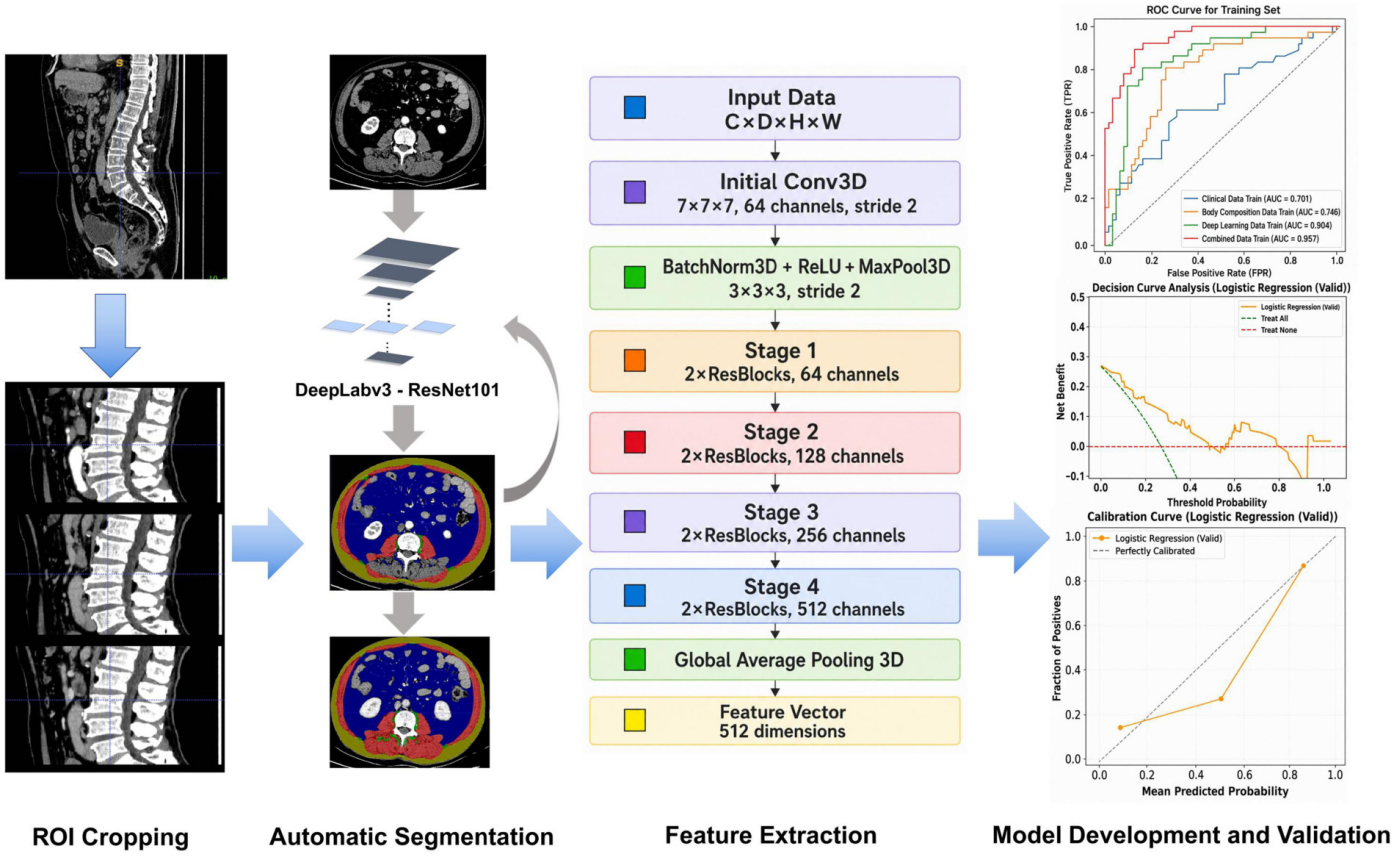
Research process.

Between January 2020 and December 2024, a total of 154 patients with pathologically confirmed CRC who underwent radical surgical resection were retrospectively enrolled from two medical centers, including 99 patients from the First People’s Hospital of Hefei (Center 1) and 55 patients from Binhu Hospital of Hefei (Center 2). Clinical and demographic information, including age and sex, was retrieved from the electronic medical record system using hospital admission identifiers. Tumor staging was determined according to the 9th edition of the TNM classification system. During model development, missing data were imputed exclusively using parameters derived from the training cohort, and the same parameters were subsequently applied to the validation cohort to avoid data leakage and maintain strict cohort independence. This study was conducted in accordance with the Declaration of Helsinki and approved by the Ethics Committee of the First People’s Hospital of Hefei (Approval No. 2025-176-01). The requirement for informed consent was waived owing to the retrospective nature of the study and the use of anonymized clinical data. The overall study workflow is presented in Figure 1.

**Figure 2:**
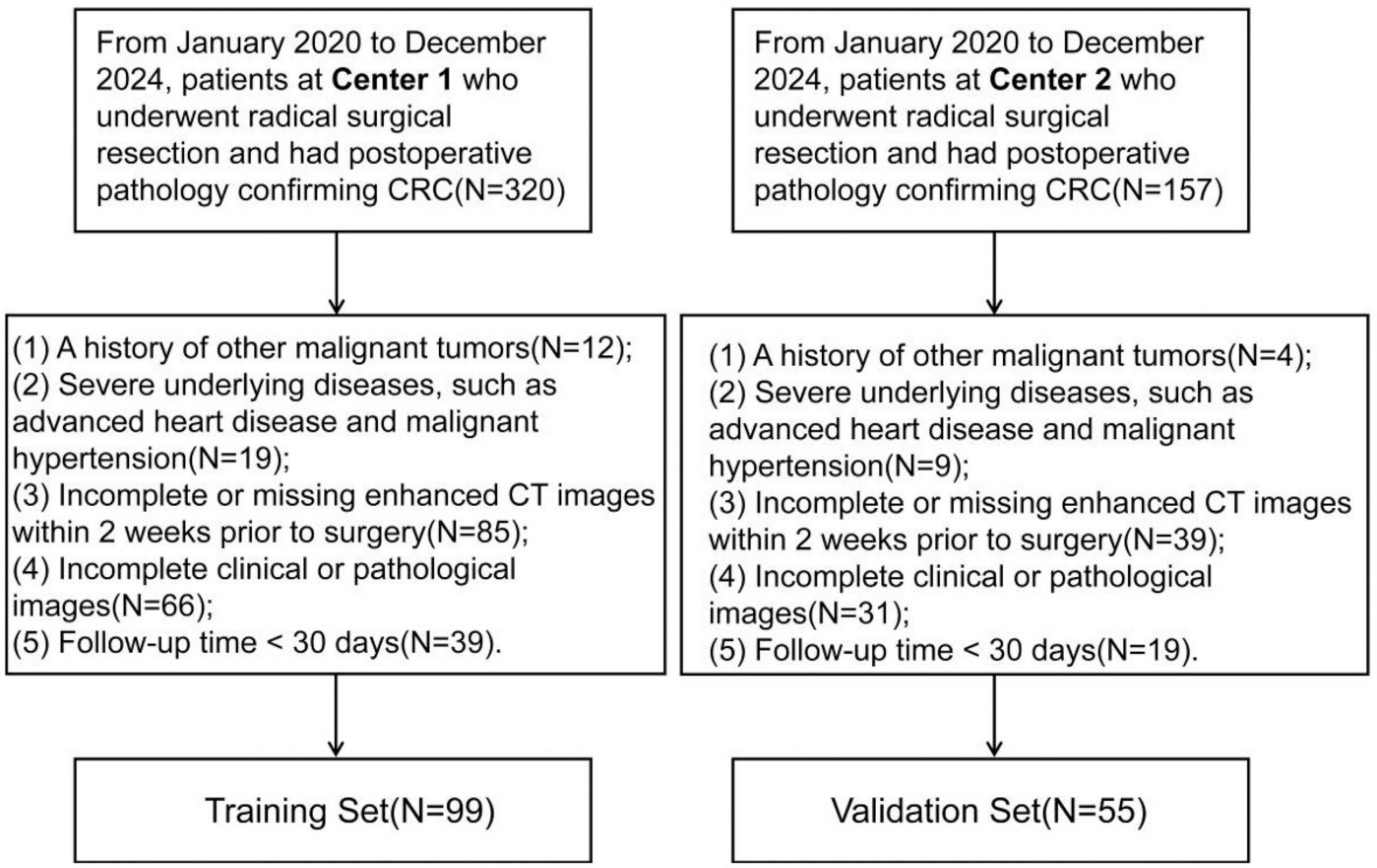
Inclusion and exclusion flowchart.

The exclusion criteria were as follows: (1) a history of other malignancies; (2) severe underlying diseases, including advanced cardiovascular disease or malignant hypertension; (3) incomplete or missing preoperative contrast-enhanced CT images acquired within 2 weeks before surgery; (4) incomplete clinical or pathological information; and (5) follow-up duration of less than 30 days. Ultimately, 154 eligible patients from the two centers were included in the study. Patients from Center 1 were assigned to the training cohort, whereas patients from Center 2 served as an independent external validation cohort. The detailed patient selection process and inclusion/exclusion criteria are illustrated in Figure 2. Postoperative complications were defined as any adverse events occurring within 30 days after radical CRC surgery and were graded according to the Clavien-Dindo classification system. Detailed definitions of postoperative complications are provided in Supplementary Appendix 1.

To minimize the risk of model overfitting and improve reproducibility, a strict data isolation strategy was implemented throughout the model development process. The external validation cohort remained completely independent and was used solely for final model evaluation, without involvement in feature selection, model training, or hyperparameter optimization procedures.

### Automated segmentation process

**Figure 3:**
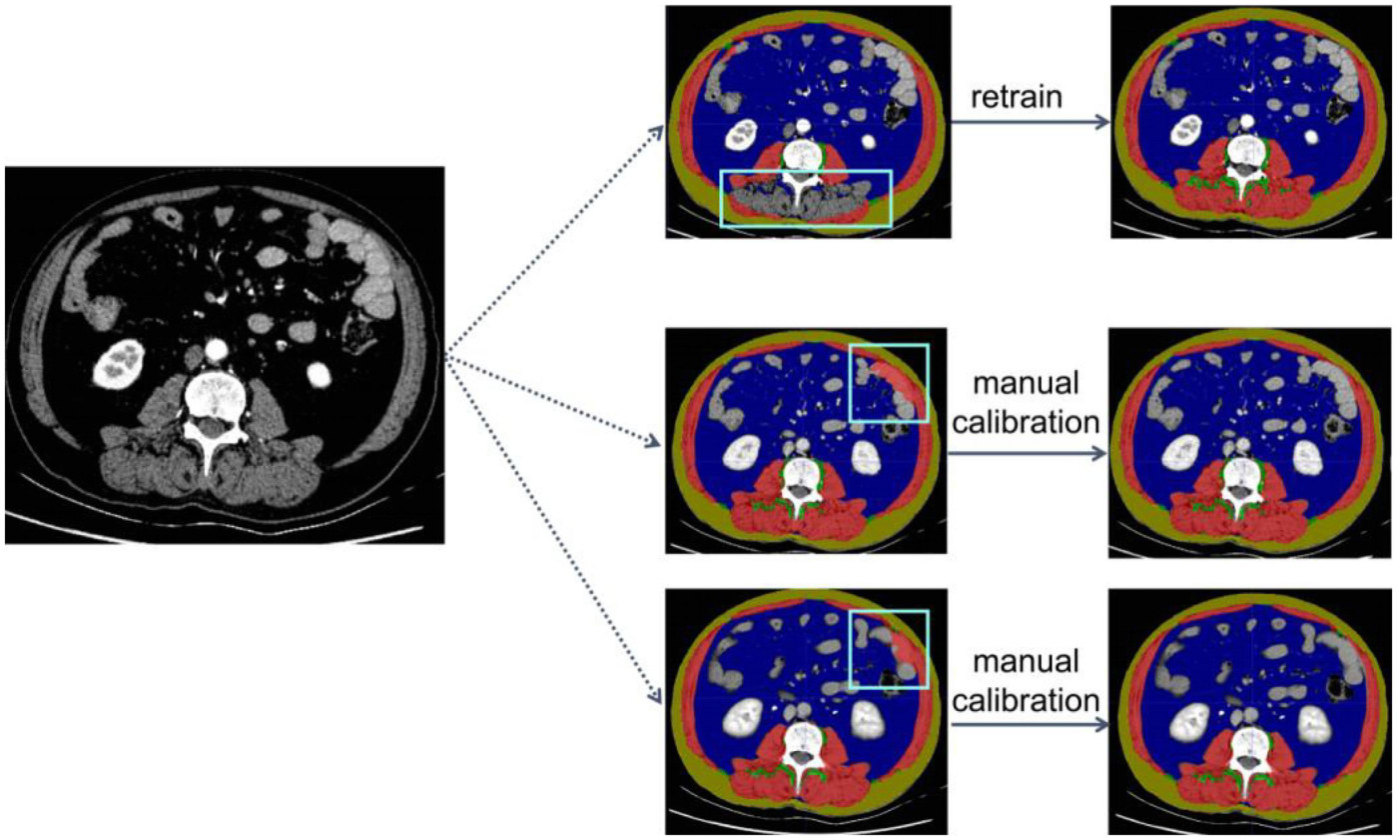
Example diagram of automatic segmentation process.

In the present study, a pretrained DeepLabv3-ResNet101 deep learning model was utilized for the automatic segmentation of regions of interest (ROI) on abdominal CT images. The model was implemented on the Onekey platform using a transfer learning strategy. Pretraining was performed on imaging data from 400 patients, yielding a Dice similarity coefficient of 0.890 (16). The automated segmentation workflow was conducted as follows. First, contrast-enhanced abdominal CT images acquired during the triphasic scanning protocol were retrieved from the Picture Archiving and Communication System (PACS). Subsequently, CT images encompassing the L1-L5 vertebral levels were extracted using the image cropping function in 3D Slicer software. This multi-slice volumetric approach was adopted to comprehensively characterize the spatial distribution and heterogeneity of body composition, thereby reducing the sampling bias commonly associated with conventional single-slice analysis methods. Next, the DeepLabv3-ResNet101 model was applied to automatically segment visceral adipose tissue (VAT), subcutaneous adipose tissue (SAT), skeletal muscle area (SMA), and intramuscular adipose tissue (IMAT), which were defined as the primary ROIs for body composition analysis. In cases where the initial segmentation results demonstrated suboptimal performance in specific regions, the corrected segmentation masks were iteratively incorporated into the model for secondary training to further improve segmentation accuracy. In addition, manual corrections were performed in regions with lower segmentation precision to remove imaging artifacts, refine tissue contours, and correct minor segmentation errors, thereby ensuring clinically acceptable segmentation quality. All manually corrected segmentation results were subsequently reviewed by an additional senior radiologist to verify the accuracy and reliability of the final data. The detailed workflow of the automated segmentation process is illustrated in Figure 3.

### Volume measurement

In the present study, a volumetric analysis algorithm was employed to quantify the volumes of VAT, SAT, SMA, and IMAT. To reduce potential measurement bias associated with a single scanning phase, volumetric measurements were independently performed on contrast-enhanced CT images acquired during the arterial, portal venous, and delayed phases, and the final values were calculated as the average across the three phases. Subsequently, all volumetric parameters were normalized by patient height to generate height-adjusted body composition indices, including VAT_h, SAT_h, SMA_h, and IMAT_h.

### Deep learning feature extraction

Given that the present study adopted a multi-slice volumetric strategy covering the L1–L5 vertebral levels, a pretrained 3D ResNet-18 model (PyTorch v2.1.0, initialized with Kinetics-pretrained weights) was used for deep learning feature extraction. The final fully connected layer outputs were extracted as feature representations. This multiphase approach allowed comprehensive characterization of tissue heterogeneity and dynamic enhancement patterns across different body composition compartments. Detailed parameter settings and feature configurations are provided in Supplementary Appendix 2. Deep learning features were obtained from VAT, SAT, SMA, and IMAT across the arterial, portal venous, and delayed phases, yielding 12 feature sets per patient, each consisting of 512 features (Supplementary Appendix 3).

### Feature selection

Feature selection was performed using a model-based feature importance strategy. The extracted deep learning features were first grouped according to the arterial, portal venous, and delayed phases. Within the training cohort, random forest, logistic regression, and extremely randomized trees algorithms combined with the SelectFromModel method were applied independently to identify informative features in each phase. Features consistently retained across multiple algorithms were considered robust candidate features. To further reduce overfitting and improve model stability, recursive feature elimination (RFE) was subsequently performed. Ultimately, the top eight features from each phase were selected, yielding a total of 24 deep learning features. These selected features were integrated to generate the DL-score, which was subsequently used for model construction and predictive analysis. The detailed calculation method of the DL-score is provided in Supplementary Appendix 4.

### Model development

In the present study, six machine learning algorithms, including logistic regression, random forest, support vector machine (SVM), k-nearest neighbors (KNN), XGBoost, and LightGBM, were constructed for predictive modeling. To optimize model performance and reduce the risk of overfitting, hyperparameter tuning was performed using the RandomizedSearchCV method. Considering the relatively limited sample size, additional regularization strategies and 5-fold cross-validation were applied during model training to improve model robustness and generalizability. Model performance was subsequently evaluated in both the training and external validation cohorts by calculating the area under the receiver operating characteristic curve (AUC), 95%CI, precision.

### Statistical analysis

Variables with missing data rates of less than 20% were included in the analysis, and missing values were imputed using the mice package in R. All statistical analyses were performed using Python (version 3.6) and R (version 4.2.2). Baseline characteristics were summarized using the R package tableone. Multivariable analyses were conducted using the CompareGroups package in R. Categorical variables were compared using Pearson’s chi-square test or Fisher’s exact test, whereas continuous variables were assessed for data distribution and compared using Student's *t*-test or the Mann–Whitney U test, as appropriate. Continuous variables were presented as mean ± standard deviation (SD) or median with interquartile range (IQR), respectively. The predictive performance of the developed models was assessed using AUC, precision. Calibration performance and clinical utility were further evaluated using calibration curves and decision curve analysis (DCA). To ensure methodological rigor and transparency, we conducted a comprehensive evaluation of model development and performance. The events per variable (EPV) was calculated based on the number of outcome events and the total number of candidate predictors included in the model to assess the potential risk of overfitting.

## Results

### Baseline characteristics

A total of 154 patients who underwent radical resection for CRC were included in the present study, comprising 99 patients in the training cohort and 55 patients in the independent external validation cohort. Comparisons of baseline clinical characteristics revealed no statistically significant differences between the two cohorts, indicating good inter-cohort comparability. Detailed baseline characteristics are summarized in Table 1. Baseline characteristics stratified by tumor location are summarized in Supplementary Table S1. In addition, body composition parameters, including SMA_h, SAT_h, VAT_h, and IMAT_h, were compared between the two cohorts. No significant differences were observed for any of these parameters. The detailed results are presented in Supplementary Table S2.

**Table 1:** Baseline table of clinical variables.

Variables	Train(*N* = 99)	Test(*N* = 55)	*P*.overall
Complications			0.332
Absent	63 (63.6%)	40 (72.7%)	
Present	36 (36.4%)	15 (27.3%)	
TNM:			0.399
T1	19 (19.2%)	14 (25.5%)	
T2	36 (36.4%)	20 (36.4%)	
T3	44 (44.4%)	20 (36.4%)	
T4	0 (0.00%)	1 (1.82%)	
Sex:			0.716
Female	44 (44.4%)	22 (40.0%)	
Male	55 (55.6%)	33 (60.0%)	
History of hypertension:			0.681
absent	57 (57.6%)	29 (52.7%)	
Present	42 (42.4%)	26 (47.3%)	
Diabetes:			0.623
Absent	82 (82.8%)	43 (78.2%)	
Present	17 (17.2%)	12 (21.8%)	
Location:			0.386
Rectum	50 (50.5%)	23 (41.8%)	
Colon	49 (49.5%)	32 (58.2%)	
NRI:			0.313
＜3	42 (42.4%)	18 (32.7%)	
≥3	57 (57.6%)	37 (67.3%)	
ASA:			0.477
Ⅰ	1 (1.01%)	0 (0.00%)	
Ⅱ	71 (71.7%)	36 (65.5%)	
Ⅲ	25 (25.3%)	19 (34.5%)	
Ⅳ	2 (2.02%)	0 (0.00%)	
Surgical Method:			0.279
Laparoscopy	71 (71.7%)	34 (61.8%)	
Open Surgery	28 (28.3%)	21 (38.2%)	
Astomosis Technique:			0.484
End to end	50 (50.5%)	21 (38.2%)	
End to side	14 (14.1%)	11 (20.0%)	
Side to side	12 (12.1%)	9 (16.4%)	
Anastomosis	23 (23.2%)	14 (25.5%)	
BMI	22.8 (2.94)	22.7 (3.50)	0.995
Age	67.2 (11.0)	67.4 (12.4)	0.914
HB	118 (20.2)	112 (24.4)	0.139
ALB	39.5 (4.11)	38.9 (6.25)	0.528
Glob	25.5 (4.22)	25.0 (4.04)	0.483
ALT	14.1 (8.24)	15.6 (14.9)	0.504
AST	19.7 (8.63)	19.0 (7.49)	0.606
CEA	7.24 (11.7)	6.53 (11.6)	0.717
OT	235 (107)	218 (81.6)	0.265
Bleed	99.4 (201)	111 (141)	0.668
Size	4.63 (1.57)	4.93 (2.25)	0.379

TNM, tumor–node–metastasis staging; NRS, nutritional risk screening; ASA, American Society of Anesthesiologists Physical Status Classification System; BMI, body mass index; HB, hemoglobin; ALB, albumin; Glob, globulin; ALT, alanine aminotransferase; AST, aspartate aminotransferase; CEA, carcinoembryonic antigen; OT, operation time.

### Feature selection

To identify potential predictors of postoperative complications in patients with CRC, univariate and multivariable analyses were performed. The results demonstrated that BMI was an independent risk factor for postoperative complications, as summarized in Table 2. Among the body composition parameters, SMA_h, SAT_h, and VAT_h showed significant associations with postoperative complications, whereas IMAT_h did not demonstrate statistical significance. Detailed statistical results are provided in Supplementary Table S3. Spearman correlation analysis showed no significant correlation between DL-score and body composition parameters (all *P* > 0.05), suggesting limited redundancy between these feature sets(Supplementary Figure S1, Table S4).

**Table 2:** Results of univariate and multivariate analysis of clinical variables.

Variables	Univariable			Multivariable		
	OR	95CI	*P*.value	OR	95CI	*P*.value
TNM						
T1						
T2	0.57	0.17–1.90	0.360			
T3	1.43	0.47–4.31	0.527			
Sex:						
Female						
Male	1.43	0.62–3.29	0.401			
History of hypertension:						
Absent						
Present	0.66	0.29–1.54	0.338			
Diabetes:						
Absent						
Present	0.48	0.14–1.60	0.234			
Location:						
Rectum						
Colon	0.51	0.22–1.17	0.113			
NRS:						
＜3						
≥3	1.26	0.55–2.90	0.591			
ASA:						
Ⅰ						
Ⅱ	33,26,736.33	0.00-Inf	0.992			
Ⅲ	32,38,769.75	0.00-Inf	0.992			
Ⅳ	57,57,812.88	0.00-Inf	0.992			
Surgical Method:						
Laparoscopy						
Open Surgery	0.77	0.31–1.95	0.584			
Astomosis Technique:						
End to end						
End to side	1.08	0.31–3.73	0.905			
Side to side	0.39	0.08–1.98	0.254			
Anastomosis	2.12	0.77–5.79	0.144			
BMI	**0**.**81**	**0.69–0.95**	**0**.**008**	**0**.**81**	**0.69–0.95**	**0**.**008**
Age	1.03	0.99–1.08	0.097	1.04	0.99–1.08	0.100
HB	1.01	0.99–1.03	0.584			
ALB	0.97	0.88–1.07	0.578			
Glob	1.03	0.94–1.14	0.534			
ALT	0.98	0.93–1.04	0.514			
AST	0.98	0.93–1.03	0.493			
CEA	0.99	0.95–1.03	0.570			
OT	1.00	1.00–1.01	0.163			
Bleed	1.00	1.00–1.00	0.211			
Size	0.96	0.74–1.25	0.764			

TNM, tumor–node–metastasis staging; NRS, nutritional risk screening; ASA, American Society of Anesthesiologists Physical Status Classification System; BMI, body mass index; HB, hemoglobin; ALB, albumin; Glob, globulin; ALT, alanine aminotransferase; AST, aspartate aminotransferase; CEA, carcinoembryonic antigen; OT, operation time.

Bold indicates the variable with *P* < 0.05 after multivariate analysis.

### Model selection

**Figure 4:**
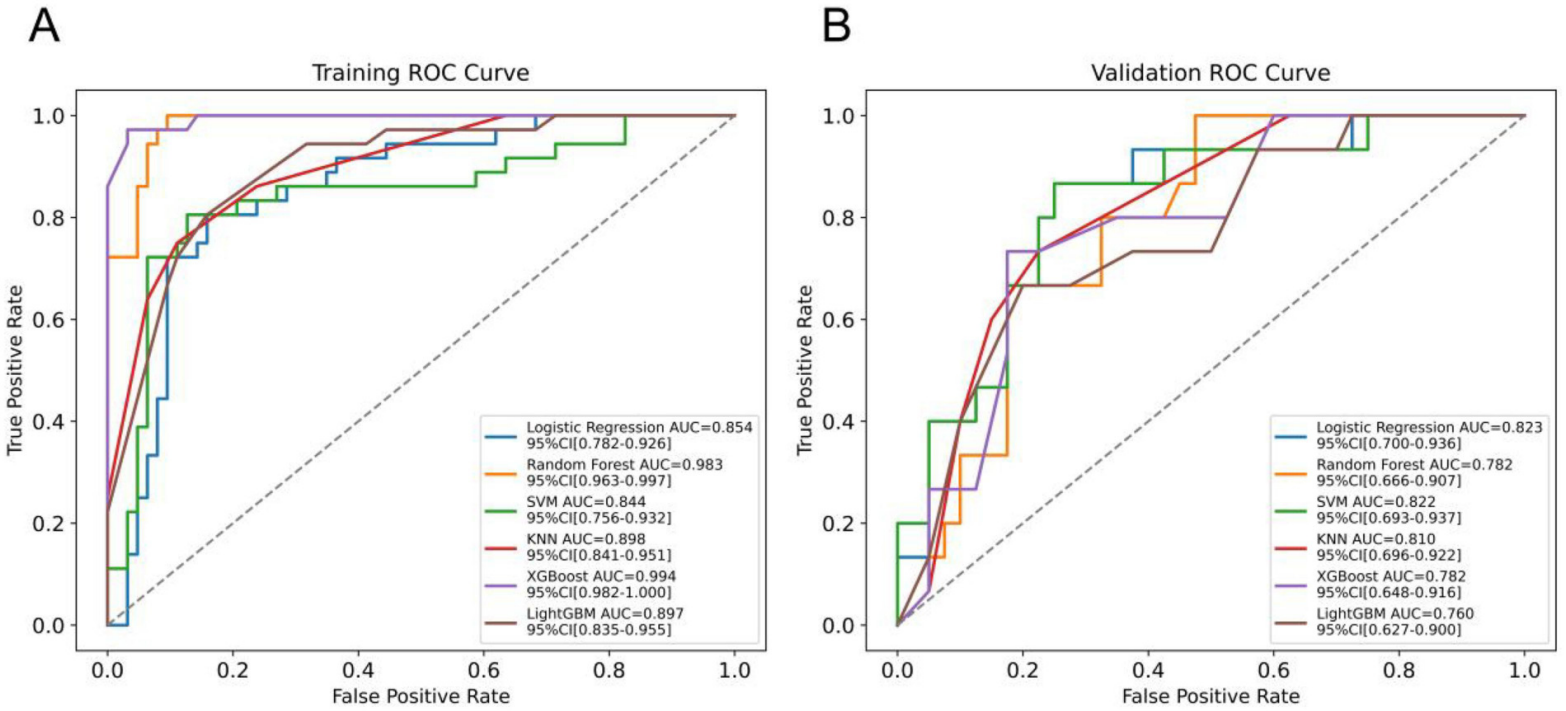
Performance of the deep learning model on six machine learning models. **(A)**: Training set, **(B)**: Independent external validation set.

The DL-score was subsequently incorporated into six machine learning algorithms, including Logistic Regression, Random Forest, SVM, KNN, XGBoost, and LightGBM, to construct predictive models for postoperative complications. The predictive performance of the different models is illustrated in Figure 4, and the corresponding precision metrics are summarized in Table 3. Among all evaluated algorithms, the Logistic Regression-based deep learning model achieved the best overall predictive performance, with AUC values of 0.854 (95% CI: 0.782–0.926) and 0.823 (95% CI: 0.700–0.936) in the training and validation cohorts, respectively. Moreover, this model yielded a precision of 0.714 in the validation cohort, outperforming the remaining machine learning models. Accordingly, Logistic Regression was selected as the optimal algorithm for subsequent model development and analysis.

**Table 3:** Performance comparison results of deep learning models.

Model	Training			Validation		
	AUC	95%CI	Precision	AUC	95%CI	Precision
Logistic Regression	**0**.**854**	**0.782–0.926**	**0**.**739**	**0**.**823**	**0.700–0.936**	**0**.**714**
Random Forest	0.983	0.963–0.997	0.909	0.783	0.666–0.907	0.500
SVM	0.844	0.756–0.932	0.778	0.822	0.693–0.937	0.588
KNN	0.898	0.841–0.951	0.794	0.810	0.696–0.922	0.600
XGBoost	0.994	0.982–1.000	0.946	0.782	0.648–0.916	0.444
LightGBM	0.897	0.835–0.955	0.744	0.760	0.627–0.900	0.556

SVM, support vector machine; KNN, k-nearest neighbors; XGBoost, extreme gradient boosting; LightGBM, light gradient boosting machine.

The bolded row in the table indicates the model (logistic regression) with better predictive performance.

### Model development and evaluation

**Figure 5:**
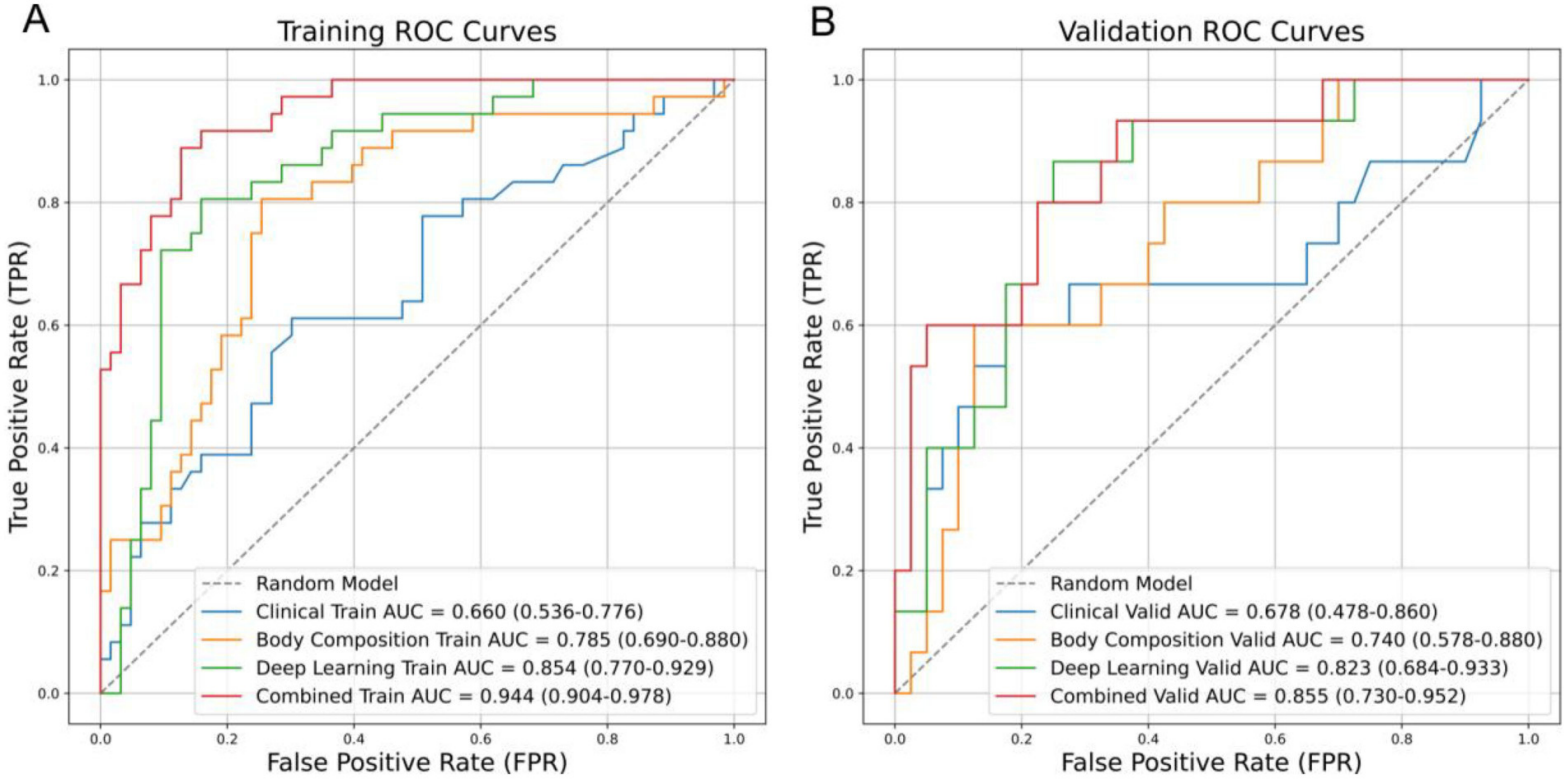
Clinical feature model, body composition model, deep learning model, and a combined model incorporating these three features established using the logistic regression algorithm. **(A)** Training set, **(B)**: Independent external validation set.

To further evaluate the predictive value of different feature categories, BMI was incorporated as a clinical variable, SMA_h, SAT_h, and VAT_h were included as body composition variables, and the DL-score was used as the deep learning feature variable. EPV was calculated based on the 36 positive events in the training cohort. The clinical model and deep learning model each demonstrated an EPV of 36, the body composition model had an EPV of 12, and the combined model showed an EPV of 7.2. Based on these variables, four Logistic Regression models were constructed, including the clinical model, body composition model, deep learning model, and combined model. The predictive performance of the four models is presented in Figure 5.

Among the developed models, the clinical model constructed using BMI alone demonstrated relatively limited predictive performance, with AUC values of 0.660 (95% CI: 0.536–0.776) and 0.678 (95% CI: 0.478–0.860) in the training and validation cohorts, respectively. After incorporating body composition parameters, including SMA_h, SAT_h, and VAT_h, the predictive performance improved, yielding AUC values of 0.785 (95% CI: 0.690–0.880) and 0.740 (95% CI: 0.578–0.880) in the training and validation cohorts, respectively. The deep learning model based on the DL-score achieved superior predictive performance, with AUC values of 0.854 (95% CI: 0.770–0.929) in the training cohort and 0.823 (95% CI: 0.684–0.933) in the validation cohort. Notably, after integrating clinical variables, body composition features, and deep learning features, the combined model demonstrated the best overall predictive performance, achieving AUC values of 0.944 (95% CI: 0.904–0.978) and 0.855 (95% CI: 0.730–0.952) in the training and validation cohorts, respectively.

To further compare the predictive performance of the four models, DeLong tests were performed in the validation cohort, and the detailed results are summarized in Table 4. No statistically significant difference was observed between the clinical model and the body composition model(*P* = 0.0898). In contrast, both the deep learning model and the combined model significantly outperformed the clinical and body composition models (all *P* < 0.05).

**Table 4:** Delong test results of the four models on the validation set.

Model 1	Model 2	Z-value	*P*-value
Clinical	Body Composition	−1.6965	0.0898
Clinical	Deep Learning Features	−4.4127	＜0.0001
Clinical	Combined Features	−3.8005	0.0001
Body Composition	Deep Learning Features	−2.0664	0.0388
Body Composition	Combined Features	−2.2101	0.0271
Deep Learning Features	Combined Features	−0.6377	0.5237

**Figure 6:**
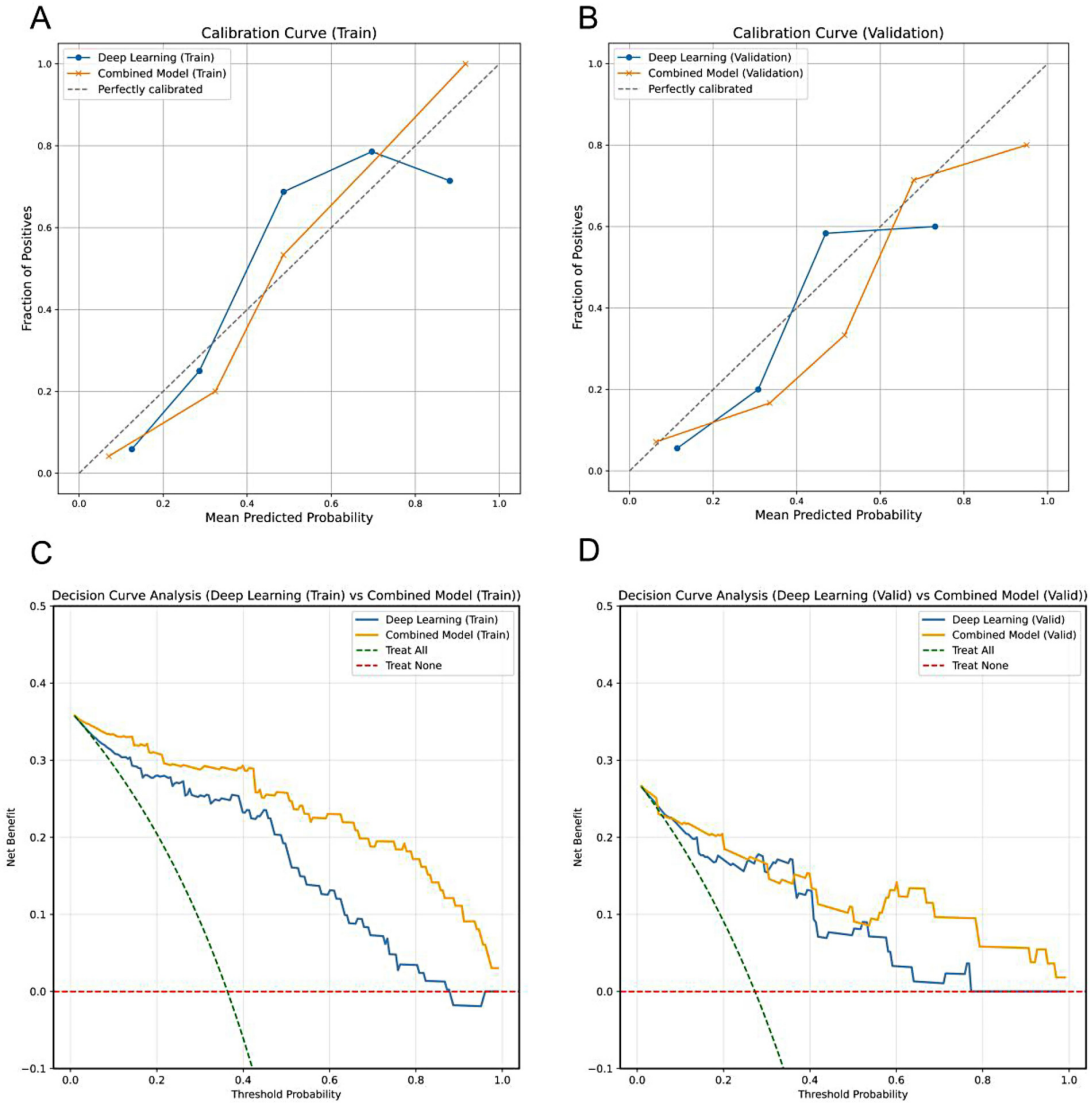
Calibration curves and DCA of the deep learning model (blue curve) and the combined model (yellow curve) on the training and validation sets. **(A,C)** show the calibration curve and DCA on the training set; **(B,D)** show the calibration curve and DCA on the validation set.

Calibration curves and DCA were subsequently performed to further assess the calibration performance and clinical utility of the deep learning and combined models. As illustrated in Figure 6, the combined model demonstrated superior calibration performance and greater clinical net benefit than the deep learning model in the training cohort. In the validation cohort, although the calibration performance of the combined model was slightly lower than that of the deep learning model, the combined model achieved higher net benefits across a wider range of decision thresholds. These findings suggest that the combined model may provide improved identification of high-risk patients while reducing unnecessary clinical interventions, thereby enhancing its overall clinical applicability.

## Discussion

Accurate prediction of postoperative complications in patients with CRC is essential for individualized perioperative management, risk stratification, and timely clinical intervention. In the present study, a deep learning-based model was developed using SMA_h, SAT_h, and VAT_h features extracted from three-phase contrast-enhanced CT images. In addition, a combined model integrating clinical variables and body composition parameters was constructed. No statistically significant difference was observed in predictive performance between the deep learning model and the combined model *P* = 0.5237. Nevertheless, compared with conventional models based solely on clinical or body composition variables, both the deep learning model and the combined model demonstrated improved predictive accuracy and enhanced clinical applicability, particularly in terms of discrimination performance and model robustness.

Following univariate and multivariate analyses, BMI was identified and incorporated as a key clinical variable in the present study. Emerging evidence has highlighted BMI as an important factor associated with postoperative complications in patients with CRC (17, 18). In particular, elevated BMI, often reflecting obesity, has been recognized as an independent risk factor for multiple adverse postoperative outcomes. A meta-analysis has demonstrated that obesity significantly increases the risk of surgical site infections following CRC surgery (19). Similarly, Liu et al. reported that higher BMI is independently associated with an increased risk of anastomotic leakage after colon cancer resection (20). These complications may contribute to prolonged hospitalization, delayed recovery, and impaired long-term prognosis and quality of life. In the present study, a clinical model incorporating BMI was developed; however, its predictive performance remained limited, with AUC values of 0.660 (95% CI: 0.536–0.776) in the training cohort and 0.678 (95% CI: 0.478–0.860) in the external validation cohort. Given the suboptimal discriminative ability of the clinical model, further incorporation of CT-derived body composition features was performed to enhance model performance and improve risk stratification capability.

Several studies have demonstrated a significant association between abnormal body composition and the occurrence of postoperative complications in patients with CRC (21, 22). In particular, AI-based analysis of CT images at the L3 vertebral level enables precise quantification of SMA, VAT, and SAT, which have been shown to predict oncological and intensive care outcomes more accurately than conventional BMI (23). However, to date, limited evidence exists regarding the use of whole-abdominal body composition analysis for predicting postoperative complication risk in CRC patients. To address this gap, the present study is the first to comprehensively incorporate body composition parameters(SMA_h, SAT_h, VAT_h, and IMAT_h) from the L1-L5 vertebral levels. Through univariate and multivariate analyses, SMA_h, SAT_h, and VAT_h were identified as independent risk factors for postoperative complications in patients with CRC (Supplementary Table S3). These findings are consistent with previous literature. For instance, Zhai et al. reported that increased visceral fat area is associated with a higher incidence of postoperative complications (24), a result further supported by a recent meta-analysis (25). The underlying mechanism may be related to the metabolic and inflammatory activity of visceral adipose tissue, which releases excessive free fatty acids and promotes macrophage infiltration and chronic systemic inflammation (26). In addition, dysregulation of adipokines may play an important role, characterized by reduced anti-inflammatory adipokines such as adiponectin and increased pro-inflammatory mediators, including interleukin-6, tumor necrosis factor-α, and C-reactive protein (27). Collectively, these alterations may exacerbate the surgical stress response and contribute to postoperative complications, such as infections and anastomotic leakage. Similarly, SMA has been shown to be independently associated with postoperative anastomotic leakage in patients undergoing CRC surgery (28). Furthermore, a systematic review and meta-analysis reported that sarcopenia is significantly associated with an increased risk of overall postoperative complications (23 studies, OR=1.84; 95% CI: 1.35–2.49), severe complications (OR=1.72; 95% CI: 1.10–2.68), and postoperative mortality (OR=3.21; 95% CI: 2.01–5.11) ^(^8). Based on these key body composition variables, a dedicated body composition-based predictive model was developed in the present study. This model achieved AUC values of 0.785 (95% CI: 0.690–0.880) in the training cohort and 0.740 (95% CI: 0.578–0.880) in the external validation cohort. Compared with the BMI-only model, the body composition model demonstrated improved predictive performance; however, the difference did not reach statistical significance.

To further improve predictive performance, the present study employed the DeepLabv3-ResNet101 architecture for automatic segmentation of regions of interest (ROIs) on abdominal CT images, followed by feature extraction using a pretrained 3D ResNet-18 network. After feature selection, six machine learning algorithms, including Logistic Regression, Random Forest, SVM, KNN, XGBoost, and LightGBM, were constructed and compared. Among these, the Logistic Regression model demonstrated the most stable and robust performance across both the training and validation cohorts (Figure 4). In contrast, more complex ensemble methods, such as Random Forest, achieved markedly higher performance in the training cohort (AUC = 0.983; 95% CI: 0.963–0.997) but exhibited a substantial decline in the validation cohort (AUC = 0.782; 95% CI: 0.666–0.907), suggesting a tendency toward overfitting under limited sample conditions. This phenomenon may be attributed to the fact that complex ensemble models are more prone to learning dataset-specific noise and spurious patterns in small samples, thereby compromising their generalizability to independent cohorts.

The results of the present study further demonstrated that the deep learning-based model achieved significantly superior predictive performance in the validation cohort compared with both the clinical and body composition-based models (*P* < 0.05). This improvement can be attributed to the ability of deep learning frameworks, particularly convolutional neural networks, to automatically extract high-dimensional and abstract imaging features, thereby capturing complex spatial heterogeneity in body composition distribution (29). Compared with conventional single-slice CT analysis, the 3D deep learning model provides a more comprehensive representation of subtle regional variations across the abdomen. Consequently, this approach not only enhances feature extraction efficiency but also improves the accuracy and robustness of postoperative complication prediction.

Further analysis demonstrated that the combined model, integrating clinical variables, body composition parameters, and deep learning-derived features, achieved the best overall predictive performance among all models evaluated. The AUC values of the combined model were 0.944 (95% CI: 0.904–0.978) in the training cohort and 0.855 (95% CI: 0.730–0.952) in the validation cohort. Although no statistically significant difference was observed between the combined model and the deep learning model in the external validation cohort, DCA indicated that the combined model provided greater net clinical benefit across a wider range of threshold probabilities. These findings suggest that the combined model may offer improved clinical decision support by enabling more accurate and individualized risk stratification.

Overall, the combined model demonstrated robust performance in both the training and external validation cohorts, indicating favorable stability and generalizability. By enabling quantitative assessment of CT-derived body composition and deep learning features, this model provides an objective and reproducible tool for preoperative risk stratification in clinical practice. From a clinical perspective, it may assist in identifying patients at high risk of postoperative complications and support individualized perioperative management strategies, including preoperative nutritional optimization, prehabilitation, intensified postoperative surveillance, and tailored surgical planning, thereby potentially improving overall clinical outcomes. Nevertheless, several limitations should be acknowledged. First, the retrospective design may introduce inherent selection bias. Second, the follow-up period was limited to 30 days postoperatively, precluding assessment of long-term outcomes. Third, although multicenter data were included to enhance cohort heterogeneity, variations in CT acquisition protocols and perioperative management strategies across institutions may still have affected feature consistency and model generalizability. Despite standardized preprocessing procedures implemented to mitigate these effects, residual inter-center variability could not be completely eliminated. Therefore, prospective multicenter studies with larger sample sizes and harmonized imaging protocols are warranted to further validate the robustness and clinical applicability of the proposed model.

## Conclusion

The combined model integrates deep learning-based analysis of CT images with clinical variables and body composition parameters, providing a comprehensive and non-invasive approach for perioperative risk stratification in patients with colorectal cancer. By enabling more accurate prediction of postoperative complications, the model may facilitate individualized perioperative management and support timely, targeted interventions for high-risk patients. Overall, this approach demonstrates promising potential as a decision-support tool for precision medicine and may contribute to improved clinical decision-making and patient outcomes in routine surgical practice.

## Data Availability

The original contributions presented in the study are included in the article/Supplementary Material, further inquiries can be directed to the corresponding author.
