# Supplementary material for: Deep learning and body composition model for predicting postoperative complications in colorectal cancer
Source: Front Med Technol. 2026 Jun 29;8:1817439. doi: 10.3389/fmedt.2026.1817439 (PMC13357902; doi:10.3389/fmedt.2026.1817439)
Supplement: Supplementary file 1 [file Supplementaryfile1.docx]

### Appendix

**Appendix 1. Standardized Definition and Classification of Postoperative Complications.**

**Appendix 2. Deep Learning Feature Extraction Based on Multi-Compartment and Multi-Phase CT Imaging.**

**Appendix 3. Detailed Procedure for Feature Extraction Using the 3D ResNet-18 Deep Learning Network.**

**Appendix 4. DL-Score Construction Based on Multi-Compartment and Multi-Phase CT Imaging.**

**Supplementary Figure S1. Spearman correlation analysis between DL-score and body composition variables**

**Supplementary Table S1. Baseline Characteristics of Patients with Tumors at Different Sites.**

**Supplementary Table S2. Baseline volume table for four types of post-body calibration in higher education institutions.**

**Supplementary Table S3. Univariate and multivariable analysis of baseline variables associated with four types of post-body calibration in higher education institutions**

**Supplementary Table S4. Spearman correlation analysis between DL-score and body composition variables**

**Appendix 1. Standardized Definition and Classification of Postoperative Complications**

Postoperative complications were defined as any adverse clinical events occurring within 30 days after radical surgery for colorectal cancer (CRC), regardless of whether the patient remained hospitalized or had been discharged. All complications were systematically identified through review of electronic medical records and postoperative follow-up data.The severity of complications was graded according to the Clavien-Dindo classification system. For analytical purposes, all complications were recorded in a standardized manner and included but were not limited to the following categories:anastomotic leakage, anastomotic stenosis, anastomotic bleeding, postoperative intra-abdominal hemorrhage, intra-abdominal infection, surgical site infection, urinary tract infection, postoperative ileus or bowel obstruction, lymphatic leakage, delayed gastric emptying, deep vein thrombosis, and other clinically relevant perioperative adverse events requiring medical or surgical intervention.For patients experiencing multiple complications, the outcome was dichotomized as the presence or absence of postoperative complications, and each patient was counted only once in the final statistical analysis.

**Appendix2. Deep Learning Feature Extraction Based on Multi-Compartment and Multi-Phase CT Imaging**

At the patient level, deep learning features were extracted separately from four anatomical compartments, including visceral fat, subcutaneous fat, skeletal muscle, and intramuscular fat, as well as from three contrast-enhanced CT phases (arterial phase, portal venous phase, and delayed phase). All extracted features were subsequently subjected to intensity normalization to minimize variations arising from different scanning conditions and acquisition settings. As a result, each patient yielded 12 distinct feature sets (4 anatomical compartments × 3 imaging phases), enabling a comprehensive characterization of tissue composition and dynamic enhancement patterns.These 12 feature sets were then concatenated in a predefined and consistent order at the patient level to construct a unified high-dimensional feature representation. This strategy enabled integrated encoding of multi-compartment and multi-phase information, thereby providing a more comprehensive description of tissue heterogeneity and imaging phenotypes across different anatomical regions.

**Appendix3. Detailed Procedure for Feature Extraction Using the 3D ResNet-18 Deep Learning Network**

In the deep learning feature extraction stage, a 3D ResNet-18 network was employed to perform end-to-end representation learning on three-dimensional CT volumetric data, aiming to effectively capture both spatial structural information and local–global contextual features within medical images. Specifically, the input consisted of preprocessed 3D CT volumes, including standardized resampling and intensity normalization, which were then resized to a fixed spatial dimension before being fed into the network.A 3D ResNet-18 architecture was adopted as the feature extraction backbone. Through its residual learning framework, the network progressively extracts multi-scale spatial features, which helps mitigate the vanishing gradient problem and enhances representational capacity in deep networks. Structurally, the final fully connected classification layer of the original 3D ResNet-18 was removed, while the network was retained up to the global average pooling layer, which served as the feature aggregation and output layer.After multiple stages of 3D convolution and residual block processing, the resulting feature maps were compressed via global average pooling into a fixed-length vector representation. Consequently, each patient’s CT volume was encoded into a 512-dimensional deep feature vector, providing a high-level semantic representation that integrates global structural and contextual information of the imaged region. This feature representation was subsequently used for downstream feature selection and predictive model construction.

**Appendix4. DL-Score Construction Based on Multi-Compartment and Multi-Phase CT Imaging**

For DL-score construction, 24 deep learning features were extracted from the penultimate layer of a pretrained 3D ResNet-18 using multi-phase (arterial, portal venous, and delayed phases) and multi-compartment CT images. Features were standardized using z-score normalization. Feature selection and weighting were performed using LASSO logistic regression, with the regularization parameter determined by 5-fold cross-validation based on minimum deviance. The DL-score was defined as a linear combination of features with non-zero LASSO coefficients. All analyses were performed in Python using scikit-learn with fixed random seeds to ensure reproducibility.

1. **Feature standardization**

All features were standardized using z-score normalization:

**X* = (X − mean) / standard deviation**

where **X** denotes the original feature value, and mean and standard deviation were calculated from the training cohort.

1. **LASSO logistic regression model**

A penalized logistic regression model with L1 regularization was applied. The model minimizes a penalized negative log-likelihood function composed of a likelihood term and an L1 penalty term:

**L(β) = negative log-likelihood + λ × sum of absolute values of regression coefficients**

where **β** represents model coefficients and **λ** is the regularization parameter selected using 5-fold cross-validation based on minimum deviance. The likelihood is defined based on logistic regression probabilities.

1. **Logistic regression probability model**

For each sample, the predicted probability is defined as:

**p = 1 / (1 + exp(−linear predictor))**

where the linear predictor is:

**linear predictor = Σ (βj × Xj*)**

1. **DL-score construction**

The DL-score was defined as a linear combination of selected features weighted by their corresponding LASSO coefficients:

**DL-score = Σ (βj × Xj*)**

Only features with non-zero coefficients after LASSO regularization were retained in the final model.

1. **Model training and validation strategy**

The optimal **λ** was determined using 5-fold cross-validation based on minimum deviance. All model development steps were performed exclusively in the training cohort. Model performance was subsequently evaluated in an independent validation cohort to ensure unbiased estimation and avoid information leakage.

**Supplementary Figure S1: Spearman correlation analysis between DL-score and body composition variables**

**
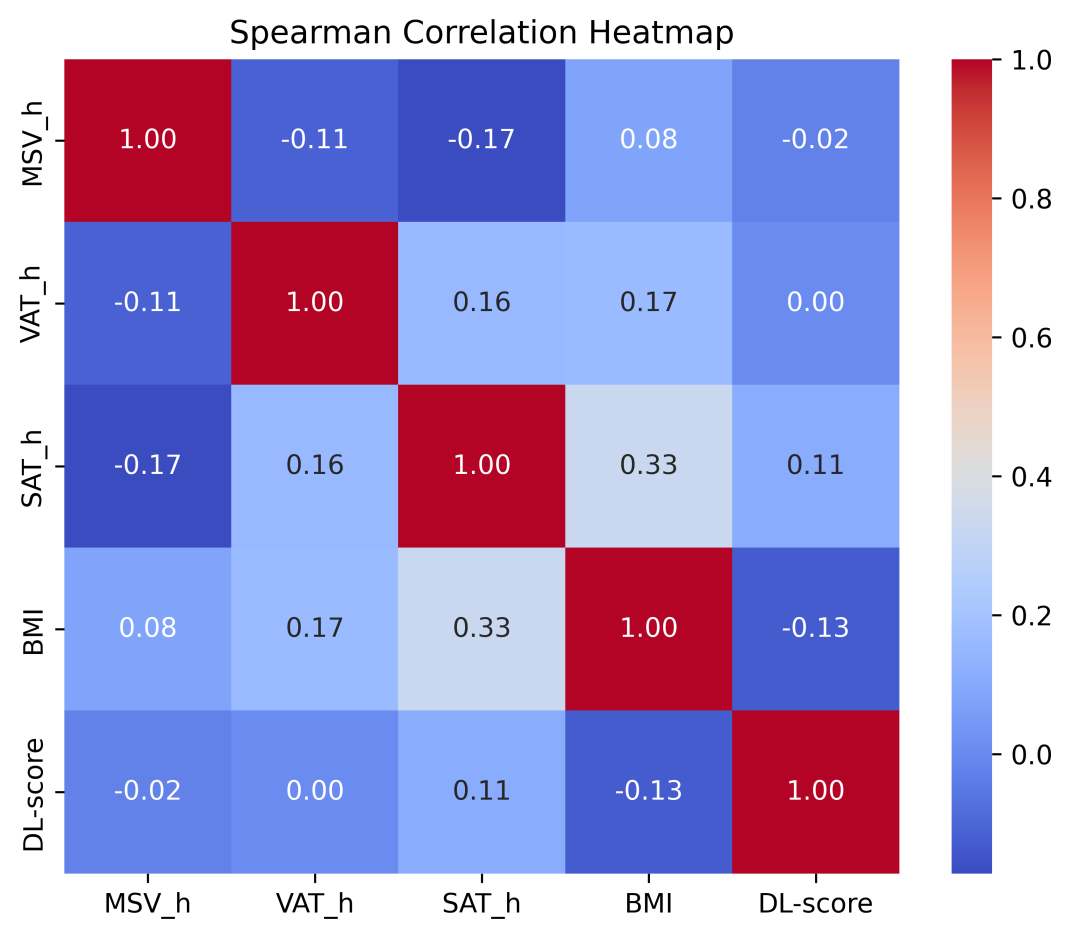
**

**Supplementary Table S1. Baseline Characteristics of Patients with Tumors at Different Sites**

|  | Rectum(N=73) | Colon(N=81) | P.overall |
| --- | --- | --- | --- |
| Complications |  |  | 0.254 |
| absent | 45 (61.6%) | 58 (71.6%) |  |
| present | 28 (38.4%) | 23 (28.4%) |  |
| TNM: |  |  | 0.083 |
| T1 | 18 (24.7%) | 15 (18.5%) |  |
| T2 | 20 (27.4%) | 36 (44.4%) |  |
| T3 | 35 (47.9%) | 29 (35.8%) |  |
| T4 | 0 (0.00%) | 1 (1.23%) |  |
| sex: |  |  | 0.043 |
| Female | 38 (52.1%) | 28 (34.6%) |  |
| Male | 35 (47.9%) | 53 (65.4%) |  |
| History of hypertension: |  |  | 0.931 |
| absent | 40 (54.8%) | 46 (56.8%) |  |
| present | 33 (45.2%) | 35 (43.2%) |  |
| diabetes: |  |  | 0.607 |
| absent | 61 (83.6%) | 64 (79.0%) |  |
| present | 12 (16.4%) | 17 (21.0%) |  |
| NRS: |  |  | 0.179 |
| ＜3 | 33 (45.2%) | 27 (33.3%) |  |
| ≥3 | 40 (54.8%) | 54 (66.7%) |  |
| ASA: |  |  | 0.073 |
| Ⅰ | 1 (1.37%) | 0 (0.00%) |  |
| Ⅱ | 56 (76.7%) | 51 (63.0%) |  |
| Ⅲ | 15 (20.5%) | 29 (35.8%) |  |
| Ⅳ | 1 (1.37%) | 1 (1.23%) |  |
| Surgical Method: |  |  | 1.000 |
| Laparoscopy | 50 (68.5%) | 55 (67.9%) |  |
| Open Surgery | 23 (31.5%) | 26 (32.1%) |  |
| Astomosis Technique: |  |  | <0.001 |
| End to end | 39 (53.4%) | 32 (39.5%) |  |
| End to side | 3 (4.11%) | 22 (27.2%) |  |
| Side to side | 0 (0.00%) | 21 (25.9%) |  |
| anastomosis | 31 (42.5%) | 6 (7.41%) |  |
| BMI | 22.5 (3.23) | 23.0 (3.06) | 0.352 |
| age | 67.3 (9.88) | 67.2 (12.8) | 0.966 |
| HB | 123 (15.9) | 109 (24.3) | <0.001 |
| ALB | 40.5 (5.09) | 38.3 (4.62) | 0.005 |
| Glob | 25.7 (4.32) | 24.9 (3.98) | 0.237 |
| ALT | 15.4 (9.00) | 14.0 (12.7) | 0.399 |
| AST | 21.1 (9.24) | 18.1 (6.94) | 0.025 |
| CEA | 7.79 (13.9) | 6.26 (9.12) | 0.428 |
| OT | 255 (118) | 205 (70.7) | 0.002 |
| Bleed | 128 (229) | 81.4 (121) | 0.120 |
| size | 4.39 (1.35) | 5.04 (2.15) | 0.025 |

**Supplementary Table S2. Baseline volume table for four types of post-body calibration in higher education institutions.**

| Variables | Training(N=99) | Validation(N=55) | P.value |
| --- | --- | --- | --- |
| MSV_h | 979 (218) | 1013 (236) | 0.39 |
| IMAT_h | 94.0 (44.1) | 79.9 (43.1) | 0.057 |
| SAT_h | 1134 (710) | 1052 (684) | 0.482 |
| VAT_h | 1541 (655) | 1435 (651) | 0.333 |

|  |
| --- |

**Supplementary Table S3. Univariate and multivariable analysis of baseline variables associated with four types of post-body calibration in higher education institutions**

|  | Univariable | | |  | Multivariable | | |
| --- | --- | --- | --- | --- | --- | --- | --- |
| Variables | OR | 95CI | P.value |  | OR | 95CI | P.value |
| MSV_h | 1.00 | 0.99-1.00 | 0.006 |  | 1.00 | 0.99-1.00 | 0.015 |
| IMAT_h | 1.00 | 0.99-1.00 | 0.633 |  | 1.00 | 0.99-1.00 |  |
| SAT_h | 1.00 | 1.00-1.00 | 0.007 |  | 1.00 | 1.00-1.00 | 0.012 |
| VAT_h | 1.00 | 1.00-1.00 | 0.003 |  | 1.00 | 1.00-1.00 | 0.007 |

**Supplementary Table S4:Spearman correlation analysis between DL-score and body composition variables**

| Variable | Spearman_r | P_value |
| --- | --- | --- |
| MSV_h | -0.021 | 0.8372 |
| VAT_h | 0.003 | 0.9779 |
| SAT_h | 0.11 | 0.2771 |
| BMI | -0.128 | 0.2053 |
